# The effect of intestinal flora metabolites on macrophage polarization

**DOI:** 10.1016/j.heliyon.2024.e35755

**Published:** 2024-08-03

**Authors:** Hengzhong Lun, Peilong Li, Juan Li, Fenfen Liu

**Affiliations:** a Department of Clinical Laboratory, The Affiliated Taian City Central Hospital of Qingdao University, Taian, Shandong, China; b Department of Clinical Laboratory, The Second Hospital, Cheeloo College of Medicine, Shandong University, Jinan, Shandong, China; c Department of Nephrology, The Affiliated Taian City Central Hospital of Qingdao University, Taian, Shandong, China

**Keywords:** Macrophage, Intestinal flora metabolites, Polarization

## Abstract

Intestinal flora metabolites played a crucial role in immunomodulation by influencing host immune responses through various pathways. Macrophages, as a type of innate immune cell, were essential in chemotaxis, phagocytosis, inflammatory responses, and microbial elimination. Different macrophage phenotypes had distinct biological functions, regulated by diverse factors and mechanisms. Advances in intestinal flora sequencing and metabolomics have enhanced understanding of how intestinal flora metabolites affect macrophage phenotypes and functions. These metabolites had varying effects on macrophage polarization and different mechanisms of influence. This study summarized the impact of gut microbiota metabolites on macrophage phenotype and function, along with the underlying mechanisms associated with different metabolites produced by intestinal flora.

## 1 Macrophage polarization

Macrophages are innate immune cells found throughout the body. They can be classified into two types based on their origins: tissue-resident macrophages, which derive from progenitor cells in the yolk sac, and monocytes, which originate from hematopoietic stem cells in the bone marrow [1]. Macrophages play critical roles in pathogen clearance, removal of senescent cells, modulation of inflammatory responses, induction of adaptive immunity, and tissue repair and remodeling [2]. Upon stimulation by various factors, macrophages polarize and differentiate into classically (M1) and alternatively (M2) activated macrophages, each performing distinct functions [3,4]. M1 macrophages are typically induced by C-reactive protein, lipopolysaccharides, and specific cytokines such as IFN-γ (interferon-γ), TNF-α (tumor necrosis factor-α), and GM-CSF (granulocyte-macrophage colony-stimulating factor) [5]. M2 macrophages can be polarized by numerous stimuli, leading to different subtypes. IL-4 and IL-13 promote M2a macrophages [6,7], while immune complexes, toll-like receptor ligands, and/or IL-1R ligands induce M2b macrophages [8,9]. IL-10, transforming growth factor-β (TGF-β), and glucocorticoids facilitate the differentiation of M2c macrophages [10]. The stimulation of both TLR (Toll-like receptor) ligands and A2 adenosine receptor agonists or IL-6 can induce the differentiation of M2d macrophages, commonly known as tumor-associated macrophages [11]. Macrophages differentiate into various phenotypes and play critical roles in clinical diseases through diverse pathways. M1 macrophages secrete high levels of pro-inflammatory factors such as interleukins (e.g., IL-6, IL-12), IFN-γ, and chemokines, which help clear invading pathogens and tumor cells, address ROS (reactive oxygen species)-induced tissue injury, impede wound healing and tissue regeneration, and enhance adaptive immunity [[11], [12], [13]]. M2a macrophages facilitate tissue repair by secreting pro-fibrotic substances like TGF-β, fibronectin, and insulin-like growth factor [14,15]. M2b macrophages increase the production of TNF-α, IL-6, IL-1, and IL-10, while reducing IL-12 secretion, thereby suppressing the acute inflammatory response [16]. M2c macrophages exhibit strong anti-inflammatory and pro-fibrotic activities by secreting high levels of TGF-β and IL-10 [14,15,17]. M2d macrophages have high expression of IL-10, TGF-β, and vascular endothelial growth factor (VEGF), but low expression of IL-12, TNF-α, and IL-1β. These macrophages play key roles in the inflammatory response within tumor tissue, promoting angiogenesis and cancer metastasis [18,19]. This study will focus on the impacts of intestinal flora metabolites on macrophage polarization and the underlying mechanisms, and will explore the connection between modulation of macrophage polarization by intestinal flora metabolites and its implications in clinical diseases.

## 2 Intestinal flora metabolites regulate macrophage phenotype and function

**Fig. 1:**
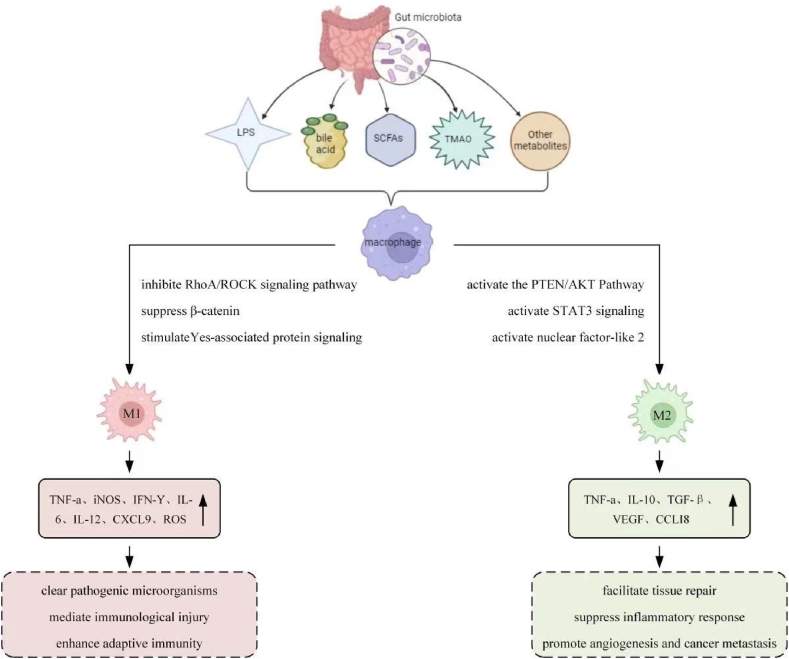
Different metabolites produced by intestinal flora stimulate macrophages to differentiate into M1 and M2 macrophages.

The intestinal microbiota is a complex community that serves as an internal ecosystem, regulating various aspects of host physiology and pathology [20]. Intestinal flora metabolize the host's food to produce short-chain fatty acids, benzoyl, choline metabolites, phenolics, bile acids, phenyl derivatives, lipids, indole derivatives, polyamines, vitamins, and other metabolites [21]. The host gut absorbs these metabolites, which function as signaling molecules to regulate host metabolism, immunity, and inflammatory responses. Intestinal flora metabolites significantly impact host health, with recent studies highlighting their role as key regulators of macrophage function and phenotype (Fig. 1).

### 2.1 Short-chain fatty acids (SCFAs)

Anaerobic bacteria in the colon ferment carbohydrates from undigested food residues to produce SCFAs. SCFAs play crucial roles in maintaining intestinal microbiota balance, preserving intestinal barrier integrity, and regulating host metabolism and immunity [22]. SCFAs include propionate, butyrate, valproic acid, and others. The bacteria responsible for SCFA production are typically part of clostridial clusters IV and XIVa within the Firmicutes phylum, including species such as *Faecalibacterium, Coprococcus, Eubacterium,* and *Roseburia* [21]. Studies have shown that different acetate derivatives affect macrophage phenotype in distinct ways. Aminooxyacetic acid administration suppressed M1 macrophage polarization and enhanced an M2-like phenotype by inhibiting the NLRP3 (NLR family pyrin domain containing protein 3)-Caspase1/IL-1β axis in myocardial infarction mice [23]. Research on butyric acid has focused on butyrate and its derivatives, both of which reduce inflammation by suppressing M1 polarization and enhancing M2 polarization in macrophages. Butyrate has been shown to inhibit M1 polarization of macrophages both in vitro and in vivo [[24], [25], [26], [27], [28]]. Cholan et al. found that butyrate decreased the recruitment of M1-type pro-inflammatory macrophages and neutrophils to zebrafish wound sites [29]. 3-Hydroxybutyrate attenuates chronic colitis and atherosclerosis by reducing the proportion of M1 macrophages in mice [30,31]. β-Hydroxybutyrate decreased M1 marker (CD16) levels and substantially elevated the M2a biomarker (Arg1), thus ameliorating inflammation after spinal cord injury by suppressing the NLRP3 inflammasome [32]. Valproic acid (VPA) exhibits various effects in different environments: it promotes M2 polarization of macrophages under normal conditions, suppresses M1 cell surface biomarker CD86 expression in systemic lupus erythematosus (SLE)-derived macrophages, and selectively induces macrophages to polarize toward the M2 phenotype in normal tissues, thereby exerting an interventional effect on tumor growth and a protective effect on normal tissues [33,34]. Another study demonstrated that VPA inhibited tumor growth by inducing myeloid-derived macrophages and polarizing them toward the M1 phenotype in a tumor growth environment [35].

### 2.2 Bile acids

Bile acids are endogenous molecules produced by the liver as byproducts of cholesterol metabolism. After synthesis in the liver, they are found in bile as various potassium or sodium salts. Bile acids promote the digestion and absorption of fats in the intestine and also regulate glucose metabolism, energy metabolism, and inflammatory responses [36]. Bile acids include cholate, glycocholate, deoxycholate, chenodeoxycholate, ursodeoxycholate, hyodeoxycholate, taurohyocholate, glycochenodeoxycholate, glycodeoxycholate and others. The bacteria involved in bile acid production include *Lactobacillus, Bifidobacteria, Enterobacter, Bacteroides,* and *Clostridium* [21]. The expression of M2 markers in bone marrow-derived macrophages (BMDM) treated with tauroursodeoxycholic acid was higher than in those treated with granulocyte-macrophage colony-stimulating factor [37]. Deoxycholic acid can enhance M1 pro-inflammatory polarization of macrophages, partially by activating toll-like receptor 2 (TLR2) via the M2 muscarinic acetylcholine receptor (M2-mAchR)/Src pathway [38]. Chenodeoxycholic acid suppresses acute myeloid leukemia progression by promoting lipid peroxidation via the ROS/p38 MAPK/DGAT1 pathway and inhibiting M2 macrophage polarization [39]. Ursodeoxycholic acid alleviates low birth weight-induced colonic inflammation by enhancing M2 macrophage polarization [40].

### 2.3 Choline metabolites

Choline is an essential nutrient for the human body, which can be synthesized endogenously in the liver or obtained exogenously from food. It is involved in regulating cellular structure, nervous system development, and fat metabolism. Choline metabolites include trimethylamine-N-oxide (TMAO), dimethylamine, betaine, and others, with related bacteria such as *Bifidobacterium* and *Faecalibacterium prausnitzii* [21]. Trimethylamine-N-oxide, a metabolite produced by intestinal flora, has been extensively studied in recent years, including its effects on macrophages. Shi et al. found that TMAO improved carotid atherosclerotic plaque stability by enhancing macrophage M2 polarization and efferocytosis [41]. However, two other studies showed that TMAO promoted M1 macrophage polarization by activating the NLRP3 inflammasome [42,43].

### 2.4 Phenolic, benzoyl, and phenyl derivatives

This category of intestinal flora metabolites includes phenyl, benzoyl, and phenolic derivatives such as hippuric acid, 2-hydroxybenzoic acid, and benzoic acid. The related bacteria include *Lactobacillus, F. prausnitzii, Bifidobacterium, Clostridium difficile,* and *Subdoligranulum* etc [21]. Currently, no studies have investigated the relationship between intestinal flora-derived phenyl, benzoyl, and phenolic derivatives and macrophage polarization. Future research could focus on how these metabolites regulate macrophage polarization.

### 2.5 Indole derivatives

Indole is a nitrogen heterocyclic compound synthesized, degraded, and transformed by microorganisms. Indole derivatives, found in high abundance in the human intestine and immune system, exhibit antioxidant and antitumor activities [44]. These derivatives include indole, indoxyl sulfate, melatonin, serotonin, and others, with related bacteria such as *Clostridium sporogenes* and *E. coli*, etc [21]. Indole-3-carbinol treatment can activate the aryl hydrocarbon receptor in macrophages, leading to increased expression of M2 biomarkers and decreased expression of M1 biomarkers in SLE patients [45]. An increased level of indole-3-acetic acid has been associated with a reduced M1/M2 ratio in the liver following sleeve gastrectomy, which could help alleviate nonalcoholic fatty liver disease in obese individuals [46]. Indoxyl sulfate induces M1 macrophage polarization in LPS-stimulated macrophages by suppressing β-catenin and activating Yes-associated protein signaling [47]. Serotonin, an indole derivative, upregulates M2 polarization-associated gene expression and downregulates M1-associated gene expression through activation of the 5-hydroxytryptamine receptor 2B and 5-hydroxytryptamine receptor 27 [48]. Melatonin regulates macrophage phenotype and function by inhibiting M1 polarization and promoting M2 polarization. It reduces inflammatory responses in diabetic trauma, spinal cord injury, and atherosclerosis through various mechanisms, thus promoting healing and functional restoration (Table 1). However, Xu et al. demonstrated that melatonin reduced choroidal neovascularization (CNV) in progressive wet age-related macular degeneration by shifting macrophage/microglia polarization from M2 to M1 phenotypes via suppression of the RhoA/ROCK signaling pathway in CNV [49].

**Table 1:** The effects of melatonin on macrophage polarization.

Disease or function	Phenotype	Mechanism
Diabetic wound healing	Increased M2/M1 ratio	Activating the PTEN/AKT Pathway [50]
Stabilize rupture-prone vulnerable plaques	Suppressed M1 polarization	Regulating the AMPKα-STATs pathway [51]
Alleviate adipocyte inflammation	Increased M2/M1 ratio	Transporting exosomal α-ketoglutarateto macrophages and promoting ten-eleven translocation mediated DNA demethylation [52]
Spinal cord injury	Enhanced M2 polarization	None [53].
Atherosclerosis	Suppressed M1 polarization and enhanced M2 polarization	Affecting inflammatory pathways that have been linked to atherosclerosis [54]
Reduce stress-induced inflammatory responses	M1 to M2 phenotype switch is induced	Activating STAT3 signaling [55]
Spinal cord injury	Increased M1 to M2 polarization	Ubiquitin-specific protease 29 interacts with deubiquitinates and stabilizes nuclear factor-like 2 [56]
Alleviate PM-triggered atherosclerosis	Alleviated M1 polarization	Regulating NOX2-mediated oxidative stress homeostasis [57]
Attenuated CNV of advanced wet age-related macular degeneration	Shifted polarization from M2 to M1	Inhibiting RhoA/ROCK signaling pathway [49]
Influenza A-Induced Acute Lung Injury.	Suppressed M1 polarization	Activating ApoE/LDLR Pathway [58]
Attenuated senescence-associated CNV.	prevented M2 polarization	inhibiting the IL-10/STAT3 pathway [59]
ameliorated osteoporosis	modulated the dynamic balance of M1/M2 macrophages	modulating SCFA and TMAO metabolism [60]

### 2.6 Vitamins

Vitamins are essential organic compounds that can be obtained from food or synthesized by intestinal bacteria. They play critical roles in metabolism, growth, development, and overall health. Vitamins include vitamin B, pyridoxine, vitamin D, and others, with related bacteria such as *Lactobacillus* and *Bifidobacterium* [21]. All-trans retinoic acid enhances the shift from M1 to M2 phenotypes in mouse strain-derived BMDMs [61]. Therapy with B vitamin complexes enhances M1 to M2 macrophage polarization in peripheral nerve injury [62,63]. Niacin, a member of the B vitamin group, has been recently shown to promote the polarization of M1 macrophages to the M2 subtype. It also plays a role in conditions such as high-fat diets, spinal cord injury, and Parkinson's disease [[64], [65], [66]]. Recent studies have focused on the regulation of macrophages by vitamin D or its analogues, such as 1,25-dihydroxyvitamin D3 [1,25(OH)₂D₃], vitamin D3, 25-hydroxyvitamin D3 [25(OH)D₃], and calcitriol. The results indicate that these compounds affect macrophage differentiation into various phenotypes (Table 2). In visceral adipose tissue, but not in subcutaneous tissue, pyridoxamine increases M2 polarization and reduces M1 polarization of macrophages [67].

**Table 2:** The effects of vitamin D or vitamin D analogues on macrophage polarization.

Disease or function	Vitamin D or its analogues	Mechanism
prevents podocyte injury in diabetic nephropathy rats	Vitamin D	Inhibit M1 macrophage activation and enhance M2 macrophage phenotype [68].
Diabetic nephropathy	Vitamin D	Inhibit macrophage transition to the M1 phenotype through suppressing STAT-1/TREM-1 pathway [69].
Epicardial adipose tissue of atherosclerotic swine	Vitamin D	Attenuate the inflammatory cytokines and promote M2 macrophages [70].
ameliorate bronchopulmonary dysplasia	Vitamin D	Regulate the balance between M1 and M2 macrophages [71].
protecte particles induced lung injury	Vitamin D	promote alveolar macrophage polarizing to M2 phenotype in a KLF4-STAT6 manner [72]
Wound microenvironment	Vitamin D3	Combining VD3 and spironolactone treatment decrease the number of local proinflammatory M1 macrophages [73].
Bone injury	1,25(OH)_2_D3	Suppress M1 macrophage-mediated enhancement of mesenchymal stem cells migration and inhibite M1 macrophage secretion of osteogenic proteins [74].
decrease the growth and migration of ovarian tumor cells	1,25(OH)_2_D3	Reverse the polarization of M2 macrophages [75].
High glucose-induced macrophage	1,25(OH)_2_D3	Promote shift from M1 to M2 macrophage via the vitamin D receptor-peroxisome proliferator-activated receptor***γ*** (VDR-PPARγ) Signaling Pathway [76].
P.aeruginosa infected macrophages	1,25(OH)_2_D3	Increase the M1/M2 ratio [77].
Type 2 diabetic	1,25(OH)_2_D3	promoted M1 phenotype switching to M2 via the VDR-PPARγ pathway [78].
RAW264.7 macrophages	1,25(OH)_2_D3	Induce macrophage polarization to M2 by upregulating T-cell Ig-mucin-3 expression [79].
Murine peritoneal macrophages	1,25(OH)_2_D3	Inhibite M1 polarization, facilitate M2 polarization [80].
ameliorates experimental inflammatory bowel disease	1,25(OH)_2_D3	Promote M1 macrophage polarization to the M2 subtype by inhibiting miR-125b [81].
Exacerbated tubulointerstitial injury in mice	25(OH)D3	Upregulate the levels of both M1 biomarker (TNF-α) and M2 biomarker (TGF-β1) in kidney-infiltrating macrophages [82].
improves diabetic wound healing	1,25(OH)D	promoted the transition of macrophages from M1 to M2 phenotype and alleviated excessive inflammation [83].
Murine bone marrow-derived macrophages	Calcitriol	Promote M2 macrophage differentiation in the presence of conditioned media from metastatic breast tumor cells [84].
increased lung metastasis in mice with 4T1 murine breast cancer	Calcitriol	promotes M2 polarization of tumor-associated macrophages via the induction of proinflammatory cytokines [85].
impact breast cancer progression	Calcitriol	inhibit M2 macrophage polarization via regulating mTOR activation in the tumor microenvironment [86].
suppresses gastric cancer progression and cisplatin resistance	Calcitriol	Inhibit and M2 macrophage polarization through inhibition of mTOR activation [87]

### 2.7 Polyamines

The small intestine absorbs polyamines, which are biologically active nitrogenous bases with low molecular weight and an aliphatic structure. They are present in many types of food and can also be produced by intestinal bacteria. Polyamines possess anti-inflammatory and immunosuppressive properties. Lowering polyamine levels can enhance the anti-tumor immune response [88]. Polyamines include putrescine, cadaverine, spermidine, and spermine, with related bacteria such as *Campylobacter jejuni* and *Clostridium saccharolyticum* [21]. Spermine inhibits M1 polarization and promotes M2 polarization of liver-resident macrophages through ATG5-dependent autophagy, leading to reduced liver injury [89]. In mice, spermine can ameliorate the degradation of articular cartilage by inhibiting ERK MAPK and p65/NF-κB signaling in macrophages, resulting in a transformation from M1 to M2 subtypes [90]. Another study suggested that spermidine might protect against collagen-induced arthritis in mice by inhibiting M1 macrophage polarization in synovial tissue [91]. Additionally, spermidine-functionalized injectable hydrogels promote the polarization of macrophages toward the regenerative M2 phenotype and enhance healing of diabetic wounds in situ [92].

### 2.8 Lipids

Lipids are a group of organic compounds that provide essential fatty acids and energy to the body. They include conjugated fatty acids, lipoproteins, cholesterol, LPS, and others, with related bacteria such as *Clostridium, Roseburia, Citrobacter, Klebsiella, Enterobacter, Lactobacillus,* and *Bifidobacterium* [21]. LPS is well-known for its strong ability to induce macrophage polarization and can stimulate classically activated macrophages with IFN-γ and LPS. Colin et al. suggested that high-density lipoprotein (HDL) does not impact the M2 polarization of human monocyte-derived macrophages; therefore, the anti-inflammatory characteristics of HDL in humans are likely unrelated to the enhancement of the M2 macrophage phenotype [93]. However, two other studies offered different perspectives: Barrett et al. showed that in a diabetic environment, HDL promoted plaque macrophage polarization toward the M2 phenotype, representing an atherosclerosis-resolving state [94]. Another study found that HDL decreased macrophage polarization to the inflammatory M1 phenotype by modifying caveolin-1 and preventing ERK1/2 and STAT3 activation [95]. Mitochondrial apolipoprotein A-I binding protein in macrophages performs an antiatherosclerotic role by regulating PINK1-dependent mitophagy and M1/M2 polarization [96]. Cholesterol affects lysosomal activity in hepatocytes, leading to M1 polarization of macrophages through exosomal miR-122-5p [97]. 27-Hydroxycholesterol induces macrophage polarization toward the M2 phenotype, which has immunomodulatory functions and helps stabilize atherosclerotic plaques. It also increases the number of total and M1-type macrophages in white adipose tissue, promoting adiposity by directly influencing white adipose tissue and triggering adipose inflammatory responses [98,99].

### 2.9 Other intestinal flora metabolites

In addition to the intestinal flora metabolites mentioned above, other metabolites can also influence macrophage polarization. Glycyrrhizic acid reduced the number of M1-like macrophages and their production of CC chemokine ligand 2 (CCL2) and tumor necrosis factor-α in the colon, thereby mitigating gut microbiota dysbiosis induced by a high-fat diet [100]. In bone marrow-derived macrophages, urolithin A promotes mitochondrial respiration and M2 polarization [101]. Creatine acids play a crucial role in repairing peripheral nerve damage by reducing M1 polarization through suppression of the JAK2/STAT1 pathway [102]. Another study showed that creatine supplementation promoted intratumor macrophages to polarize towards the M1 phenotype rather than the M2 phenotype, enhancing anti-tumor immunity [103]. M2 macrophages are hyperpolarized by extracellular succinate via SUCNR1/GPR91-mediated Gq signaling [104]. Increased intracellular succinate protects primary microglia by inhibiting their conversion to the pro-inflammatory M1 phenotype induced by LPS [105]. Warner et al. found reduced pro-inflammatory M1 macrophages and elevated T regulatory cells in the liver of ethanol-fed mice [106]. In neural tissue, alcohol feeding resulted in increased markers for both M1 and M2 microglia [[107], [108], [109]].

## 3 The impact of macrophage phenotype on clinical disease

**Fig. 2:**
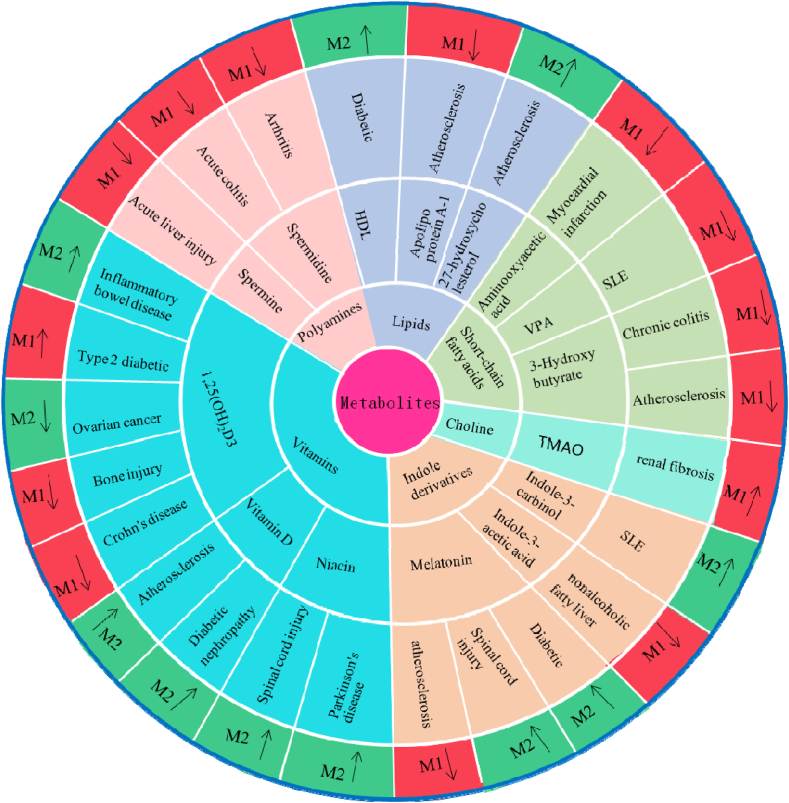
Regulation of macrophage phenotype by intestinal flora metabolites in different diseases. The circle represents different macrophage phenotypes, where red indicates M1 macrophages, green indicates M2 macrophages, and arrows indicate upregulation and downregulation, respectively.

Intestinal flora metabolites can influence clinical diseases by modulating macrophage differentiation toward the M1 or M2 phenotype, thereby either exacerbating or alleviating disease. Such effects have been studied in conditions including diabetes, atherosclerosis, and systemic lupus erythematosus (Fig. 2).

### 3.1 Macrophage polarization and tumors

Tumor-associated macrophages (TAMs) are macrophages present in the tumor microenvironment. TAMs typically polarize into two subtypes: the classically activated M1 type and the alternatively activated M2 type. The M1 type contributes to the elimination of tumor cells and defense against pathogenic invasion, while the M2 type promotes tumor growth, invasion, and metastasis. TAM differentiation is highly dependent on cancer type, stage, and tumor heterogeneity. Most TAMs are reprogrammed by the tumor microenvironment to support primary tumor growth and metastasis [110]. TAMs generally exhibit a single phenotype in breast cancer [111,112] and lung cancer [113], but in colorectal cancer, both M1 and M2 phenotypes can be present [114,115]. TAMs can promote tumor cell proliferation and metastasis by secreting various cytokines and exosomes [116,117]. Activation of the aryl hydrocarbon receptor in tumor-related macrophages by tryptophan-derived microbial metabolites suppresses anti-tumor immunity [118]. The signaling pathways implicated in the role of TAMs in tumor progression include NF-κB, TGF-β/BMPs, TNF-α/Wnt, β-catenin/Wnt, and TLR4/STAT3 [119,120]. Recent studies have shown that esophageal squamous cell carcinoma (ESCC)-derived exosomes containing hyaluronidase 1 facilitate M2 macrophage polarization by targeting Aurora B kinase to activate the PI3K/AKT signaling pathway, which in turn promotes ESCC progression [121]. Another study suggests that the low-density solute carrier family 3 member 2 (SLC3A2) induces macrophage phenotypic reprogramming through arachidonic acid in lung adenocarcinoma [122].

### 3.2 Macrophage polarization and inflammatory diseases

Macrophages are involved in intra-tissue homeostasis by either promoting or suppressing inflammation, which can lead to tissue damage or repair. Macrophage polarization is dynamic and reversible; it can shift from M1 to M2, or vice versa, in response to changes in microenvironmental conditions [123]. The pathogenesis of inflammatory bowel disease is thought to result from an imbalance in intestinal macrophage polarization, which triggers and perpetuates excessive inflammatory immune responses [124]. Lactobacillus lactis EJ-1 has been shown to inhibit NF-κB signaling and regulate the M1/M2 macrophage transition, contributing to the improvement of colitis [125]. Disturbed regulation of lung macrophage phenotype is a mechanism in asthma pathogenesis, and different polarization types of lung macrophages can influence the direction of allergic asthma development [126].

Recent studies have shown that activation of P2X7R by ATP induces M2 polarization of lung macrophages while suppressing M1 polarization, a mechanism involved in asthma development [127]. Macrophages play a crucial role in the development, progression, and regression of atherosclerosis (AS). Current research suggests that low-density lipoprotein is the primary source of lipid accumulation in atherosclerotic lesions, leading to endothelial activation and recruitment of monocytes, which differentiate into M1-type macrophages. These macrophages then engulf both unmodified and modified lipoproteins, transforming them into cholesterol-rich foam cells that contribute to lipid storage and plaque growth [128]. In AS, macrophages predominantly polarize towards the M1 phenotype, and M2 polarization is inhibited. The extent of M1 polarization correlates positively with the severity of arterial stenosis. Caidahl et al. [129] observed elevated levels of CCL19 in the serum of individuals with AS and patients with angina pectoris, along with a 130-fold increase in M1 expression compared to M2. In rheumatoid arthritis (RA), dysregulation of synovial miR-221-3p expression drives a shift from M2 to the pro-inflammatory M1 macrophage. This shift results from the inhibition of JAK3/STAT3 activation, leading to a weakened anti-inflammatory response and an increased M1/M2 ratio, which further drives RA development [130]. In periodontitis, IL-37 prevents disease progression by suppressing NLRP3 inflammasome activation and mediating M1/M2 macrophage polarization [131]. In osteoarthritis (OA), M2 macrophage-derived exosomal miR-26b-5p regulates macrophage polarization and delays OA progression by targeting TLR3 and COL10A1 [132].

### 3.3 Macrophage polarization and metabolic diseases

The polarization of macrophages is critical in the progression and treatment of metabolic diseases. Obesity, a metabolic disorder, promotes the infiltration of inflammatory macrophages into adipose tissue and a shift in macrophage phenotype from M2 to M1 [133]. Consequently, obesity may be associated with an overreaction of M1 pro-inflammatory macrophages and an imbalance in macrophage polarization. Adjusting macrophage polarization and increasing the formation of anti-inflammatory macrophages is a potential approach for treating obesity. It has been shown that spexin decreases M1 macrophage polarization directly or indirectly through mature adipocytes, thereby improving obesity [134]. In diabetic patients, pro-inflammatory cytokine expression and M1 macrophage polarization increase, while anti-inflammatory cytokine expression and M2 macrophage polarization decrease. Zhang et al. [135] demonstrated that activated vitamin D3 reduced the progression of diabetic nephropathy by suppressing the high-glucose-induced STAT-1/TREM-1 signaling pathway, decreasing macrophage adhesion and migration, and inhibiting its conversion to the M1 phenotype.

## 4 Conclusions and future perspectives

Macrophage phenotypes exhibit diverse functions and impacts on the body. Advances in studying the effects of intestinal flora metabolites on health and disease by regulating macrophages may lead to the discovery of new targets for disease diagnosis and treatment. By using gut flora metabolites to restore the balance of M1/M2 macrophage polarization and developing targeted therapies against macrophage polarization, we can potentially reduce inflammation and intervene in tumor progression. Additionally, gut flora metabolites could serve as biomarkers for diseases associated with macrophage dysfunction; for example, apolipoprotein C1 is an immunological biomarker that regulates macrophage polarization and promotes tumor metastasis[136]. However, the impact of intestinal flora metabolites on macrophages is multifaceted, and identifying specific metabolites that significantly affect macrophage polarization and disease outcomes remains a challenge. Macrophage phenotype is influenced not only by individual intestinal flora metabolites but also by a combination of these metabolites and other factors. The mechanisms by which intestinal flora metabolites regulate macrophage polarization are still not fully understood and require further investigation. Continued research in this area will enhance our understanding of how intestinal flora metabolites affect macrophage phenotype and function, offering new insights and strategies for diagnosing and treating macrophage-related diseases through manipulation of intestinal flora and its metabolites.

## Data availability statement

All relevant data and material are presented in the paper. Also, this review paper has not generated new data.

## Consent for publication

Not applicable.

## Ethics approval and consent to participate

Not applicable.

## CRediT authorship contribution statement

**Hengzhong Lun:** Writing – original draft. **Peilong Li:** Visualization. **Juan Li:** Investigation. **Fenfen Liu:** Writing – review & editing.

## Declaration of competing interest

We declare that we have no financial and personal relationships with other people or organizations that can inappropriately influence our work, there is no professional or other personal interest of any nature or kind in any product, service and/or company that could be construed as influencing the position presented in, or the review of, the manuscript entitled.
